# Influence of Temperature on Transdermal Penetration Enhancing Mechanism of Borneol: A Multi-Scale Study

**DOI:** 10.3390/ijms18010195

**Published:** 2017-01-19

**Authors:** Qianqian Yin, Ran Wang, Shufang Yang, Zhimin Wu, Shujuan Guo, Xingxing Dai, Yanjiang Qiao, Xinyuan Shi

**Affiliations:** 1 Beijing University of Chinese Medicine, No.11 of North 3rd Ring East Road, Chaoyang District, Beijing 100029, China; qianqian.yin@tigermed.net (Q.Y.); wangran2017@sina.com (R.W.); 18811795303@163.com (S.Y.); hbjlwzm@163.com (Z.W.); 18810820913@163.com (S.G.); jolly_1987@163.com (X.D.); 2 School of Traditional Chinese Medicine, Capital Medical University, No. 10 of Xitoutiao Outside Youanmen, Fengtai District, Beijing 100069, China; 3 Key Laboratory of TCM-information Engineer of State Administration of TCM, No. 11 of North 3rd Ring East Road, Chaoyang District, Beijing 100029, China

**Keywords:** multi-scale, coarse-grained, temperature, borneol, penetration enhancement

## Abstract

The influence of temperature on the transdermal permeation enhancing mechanism of borneol (BO) was investigated using a multi-scale method, containing a coarse-grained molecular dynamic (CG-MD) simulation, an in vitro permeation experiment, and a transmission electron microscope (TEM) study. The results showed that BO has the potential to be used as a transdermal penetration enhancer to help osthole (OST) penetrate into the bilayer. With the increasing temperature, the stratum corneum (SC) becomes more flexible, proving to be synergistic with the permeation enhancement of BO, and the lag time (*T_Lag_*) of BO and OST are shortened. However, when the temperature increased too much, with the effect of BO, the structure of SC was destroyed; for example, a water pore was formed and the micelle reversed. Though there were a number of drugs coming into the SC, the normal bilayer structure was absent. In addition, through comparing the simulation, in vitro experiment, and TEM study, we concluded that the computer simulation provided some visually detailed information, and the method plays an important role in related studies of permeation.

## 1. Introduction

Transdermal drug delivery systems (TDDS), some of the most common drug administration routes, have attracted a lot of attention due to their non-invasive characteristics, avoidance of first-pass metabolism, ease of dose control and absorption, and better patient compliance [1]. Therefore, the diversity of therapeutic applications involving TDDS has been widely explored by researchers in recent years. Unfortunately, poor skin permeability became a major obstacle in the development of TDDSs. In order to tackle this problem, researchers have been dedicated to investigating various chemical or physiological methods to lower the barrier generated by a highly-organized structure of stratum corneum (SC), the outermost layer of skin [2,3]. So far, one promising approach is using a permeation enhancer (PE) [4,5]. Essentials oils are an alternative option to this issue, which are widely used in the cosmetic, pharmaceutical (in antifungal and antiviral agents), medicinal, and food industries [6]. Compared to chemical PEs, natural essential oil refined from plants has proven to be less toxic and has higher permeation enhancement ability and lower cutaneous irritancy at lower concentrations; for example, Qi-Feng Yi has investigated that borneol displayed lower cytotoxicity or irritation in comparison to the well-established and standard enhancer Azone by IC_50_ values of both HaCaT keratinocytes and CCC-HSF-1 fibroblasts [7,8,9]. This makes essential oil the first preferred PE over chemical PEs.

Borneol (BO), a traditional Chinese medicine that is extracted from *Cinnamomum camphora* (L.), is a cyclic terpene alcohol that is widely used as a resuscitation-inducing agent. Studies have shown that BO is able to assist drugs in crossing various physiologic barriers, such as the blood-brain barrier, cornea, skin, and mucous membrane [10,11,12,13]. To the best of our knowledge, BO can be used as a PE as well [14,15]. Even though there are a large amount of studies focusing on the permeation enhancement ratio, the exploration of the interaction between BO and the lipid of SC, at the molecular level, is a relatively rare investigated case. Therefore, in order to study the performance of the permeation enhancement of BO, we chose a typical external drug, osthole (OST), which is extracted from the fruit of *Cnidium monnier* (L.) Cusson. OST is usually recognized as an external drug in the clinic, serving as one of the major drugs to treat several common skin diseases and it has an obvious curative effect on skin pruritus [16,17,18].

Except for some internal properties of the drug, for example the weight and lipophilic ability [19,20,21], there are other external factors to take into consideration, such as the concentration effect, and other environmental factors, including temperature, humidity, and so forth [22]. In our previous experiments, the effect of the concentration of BO on the penetration enhancement mechanism was investigated and it revealed that the presence of borneol at any concentration within the bilayer interior can fluidize the bilayer membrane, and with the increase of the concentration, the effect differed [21,23,24]. Among these factors, temperature not only affects the diffusion of the drug, but also the functions of SC associated with its fluidity resulting from the transition (from gel to liquid crystalline) of SC lipids [25,26,27]. Although the influence of temperature on transdermal penetration has been documented, its mechanism has rarely been investigated with a scientific and systematic method. Thus, the novelty of this work is that we investigated the permeation of drugs and the performance of the permeation enhancement of BO with temperature as the principal factor.

A number of technologies have been applied to the study on the interaction between the drug and SC, including some spectrum analysis [28,29,30,31,32], electron microscope [33], and thermodynamics methods (for instance, differential scanning calorimetry) [34,35], but these methods failed to demonstrate the processes of permeation and the interaction between the drugs and lipid membrane. Given this problem, a molecular dynamic (MD) simulation was selected to investigate this process [36,37]. Coarse-grained molecular dynamic (CG-MD) simulation is a popular alternative to all-atom simulation (AA) as an important tool providing sub-nm and femto-sec resolution over extended periods of time [38,39,40]. In this study, influenced by different temperatures, the penetrating processes were simulated by CG-MD simulation in mesoscale, so that the SC changes could be gathered.

An in vitro permeation experiment was conducted using vertical Franz diffusion cells with the rat skin as the SC mode, and the changes of SC were observed by transmission electron microscope (TEM) in order to investigate the influence of temperature on the permeation enhancement of BO to OST on a large scale. With this multi-scale study, the permeation enhancing mechanism of borneol could be probed at different temperatures, with the potential to provide guidance for clinical applications.

## 2. Results and Discussion

The effect of temperature is a significant influence on transdermal permeation. The following were conducted in sequence: First, influenced by increasing temperature, the SC lipid bilayer changes, thus, an initial focus on the effect of different temperatures on SC is needed; then, because both the penetration of the drug and SC structure are influenced by various temperatures, an examination of the effects of temperature on the interaction between BO or OST and SC was conducted; finally, the permeation-enhancing effect of BO on OST at various temperatures was investigated to explain the influence of increasing temperatures on the permeation enhancement of BO by CG-MD simulation, an in vitro permeation experiment, and a TEM study. Specific results are described in the following paragraphs.

### 2.1. Influence of Temperature on the Stratum Corneum Lipid Bilayer

We selected the simulation system to only contain the bilayer and solvent (80% propanediol) to probe the influence of temperature on the bilayer. The temperature was set at 273, 298, 310, 315 and 323 K around the temperature of the human body (310 K), as well as below the phase transition temperature of our bilayer model (332.9 K).

**Figure 1:**
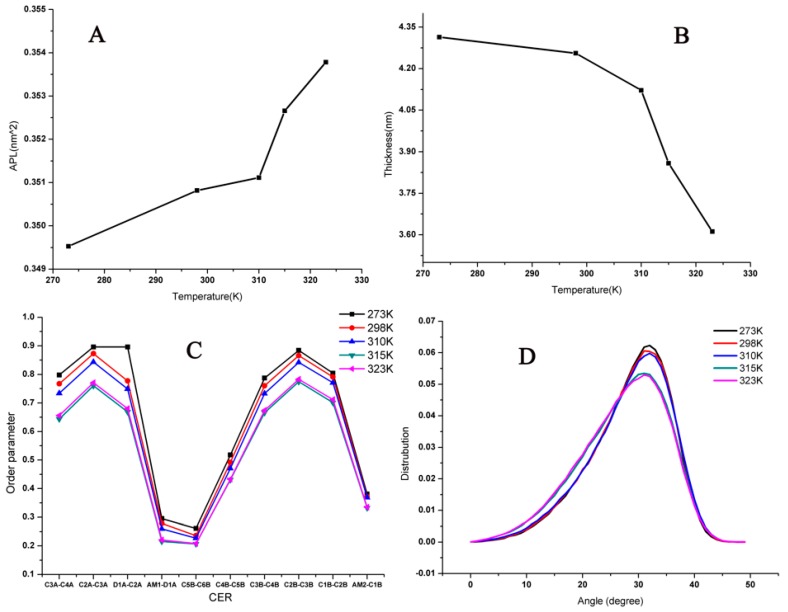
Corresponding analyses of lipid bilayer system at different temperatures: (**A**) area per lipid (APL); (**B**) thickness; (**C**) Order parameters; and (**D**) angle between two chains in the ceramides (CER).

To gain a quantitative understanding of the bilayer, some parameters were analyzed to describe the properties, including the thickness, area per lipid (APL), the order of alkyl chains, and the angle between two chains in the ceramides (CER) at different temperatures, as shown in Figure 1. It was easy to see that with temperatures increasing, the bilayer structure had no obvious changes under 310 K; however, when the temperature was increased to over 310 K, the APL went up and there was a sharp decrease in the thickness and order parameter of lipids, but the angle did not change in value. Thus, increasing temperatures influenced the distance between lipids directly instead of the angle between the two chains in the CER (these results could be identified as the lipids because they became more unconsolidated with higher temperatures.) With the increasing temperature, the bilayer became loosened, the lipids became more flexible, and there was more room for lipids to roam. The lipids became frizzier and shorter as a result of the decreased thickness and order parameter.

The influence of temperature on the bilayer was investigated, and became a crucial reference for later study.

### 2.2. Influence of Temperature on the Interaction of Borneol and Osthole with SC

Based on some quantitative structure-permeation relationship (QSPRs) model studies, drug lipophilicity is identified as the predominant physiochemical parameter that determines drug permeability. In this part, the drugs with different lipophilicity were selected (BO (logP = 2.55) and OST (logP = 3.68) to monitor the influence of temperature on the interaction between SC and the two drugs separately, and the two systems containing 10% BO and 10% OST retaining a complete bilayer structure were selected (the concentrations were set based on our previous experiment and showing in the Figures S1 and S2 in Supplementary materials [22]). During this experiment, the temperature was set at 273, 298, 310, 315 and 323 K.

**Figure 2:**
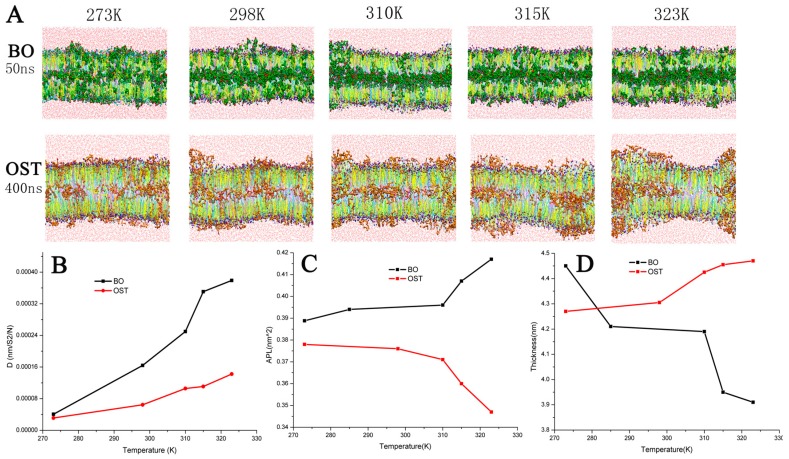
(**A**) Snapshots of balanced bilayer systems with 10% Borneol (BO) (green) or 10% Osthole (OST) (brown); (**B**) diffusion parameter of BO (black) or OST (red) at different temperature; (**C**) APL; and (**D**) thickness.

Figure 2A demonstrates the diffusion balanced status of BO and OST and the variation of the bilayer at different temperatures. When the BO system was at 50 ns and 315 or 323 K, equilibrium was reached with all of the BO inside the bilayer. At the same time, there was still a small amount of BO molecules outside the bilayer at lower temperatures. The systems with 10% OST were nearly at an equilibrium state at 400 ns. The snapshots also clearly indicated that the increasing temperature was able to assist more OST molecules to penetrate through the bilayer. Figure 2B displays the diffusion parameter of BO and OST. In A and B of Figure 2, it clearly indicates that the increasing temperature is able to assist BO or OST molecules in faster penetration through the bilayer.

Figure 2C,D show the APL and thickness of the bilayer with different temperatures, separately. After treatment with BO, the APL was much higher and the thickness was lower. Coupled with the influence of temperature on SC, APL rose and the thickness decreased drastically in the BO system. In the OST system, the APL was enhanced and the thickness was lower. In contrast, although also due to the effect of increasing temperature, the APL was reduced and the thickness was increased. This result reflected the different effects of various temperatures on the penetration of BO or OST. When the temperature was below 310 K, the increasing temperature enhanced the diffusion of OST into bilayers. When the temperature was over 310 K, the effect of the temperature was shown to promote the interaction of OST with lipids. Since the groups methoxyl and lactone in OST can be considered the hydrogen bond donors to form a firm hydrogen-bonded network with the head group, the more OST that was around the lipid, the steadier the H-bond network was present. The bilayer fluctuated violently, and the APL and thickness changed greatly.

In conclusion, either BO or OST could enhance the APL and reduce the thickness of the bilayers. When the temperature was elevated, the interactions of BO and OST with the bilayers were enhanced.

### 2.3. Influence on the Permeation Enhancing Effect of Borneol to Osthole

In order to investigate the impact of temperature on the permeation enhancement of BO to OST, CG-MD simulation, an in vitro permeation experiment, and a TEM study were selected.

#### 2.3.1. Coarse-Grained Molecular Dynamic Simulation

**Figure 3:**
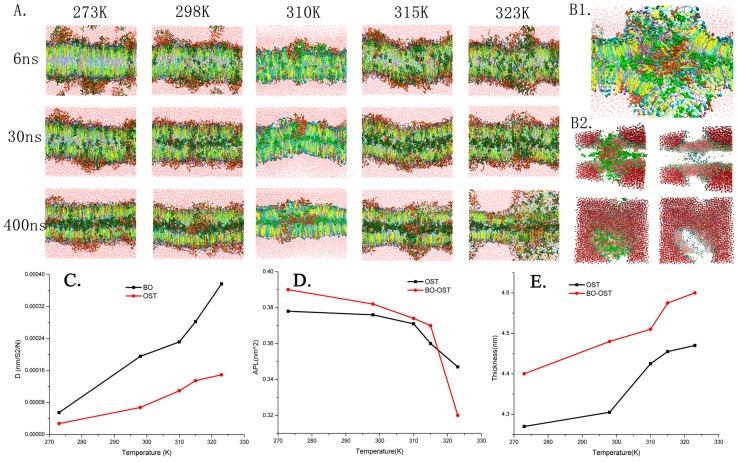
Morphology of lipid bilayer systems at different temperatures: (**A**) snapshots of the bilayer with 10% OST and 5% BO at 273, 298, 310, 315 and 323 K; (**B**) detailed structures of water pore and reversed micelle form at 323 K in 400 ns; (**C**) diffusion parameter; (**D**) APL; and (**E**) thickness.

A system containing 10% OST as the drug and 5% BO as the PE was adopted and the concentration of BO and OST were set to maintain the whole bilayer structure (showing in the Figure S3 in Supplementary materials) [22]. In this scenario, the system was still set at temperatures of 273, 298, 310, 315 and 323 K. To demonstrate the morphologic structure of all of the systems at different temperatures, several snapshots of the results are presented in Figure 3A. As shown in the figure, with the increase of temperature, the physical structure of the lipid bilayer underwent changes, such as fluctuation, bend, and deformation. At 323 K, when a water pore and reversed micelle appeared (see Figure 3B), which could be found in the system containing a high percent of BO at 310 K, as verified in our previous work [19]. For example, with the same concentration of OST (10%), the system with 10% BO at 310 K, a water pore, and reversed micelle arose; when the temperature increased to 323 K, 5% BO caused the formation of a water pore and reversed micelle. This also means that the rising temperature could enhance the effect of BO on the bilayers because of its effect on both SC structure and the penetration of the drug, which had been proven in previous experiments [21].

The plots of diffusion parameters of BO and OST, APL, and thickness of the bilayer were calculated as shown in Figure 3. When treated by BO, the diffusion of OST improved significantly, which showed the enhancement effect of BO to OST. When the temperature increased, the diffusion parameter of BO and OST were enhanced sharply. This was true especially in comparison to the system only with OST; it was obvious that both the increasing temperature and BO as PE played important roles in enhancing penetration of OST. On the other hand, except for the system at 323 K, in which the reversed micelle took shape, the APL became much higher as a result of BO’s enhancement effect. Since there were more molecules getting into the bilayer, the thickness was increased.

To sum up, BO has the potential to be used as a transdermal penetration enhancer. The increasing temperature can appropriately help BO and OST permeate and change the bilayer’s morphological characteristics, which can help effectively strengthen the permeation enhancement of BO to OST. In order to verify and complete our research, we have performed in vitro permeation studies.

#### 2.3.2. In Vitro Permeation Studies

**Figure 4:**
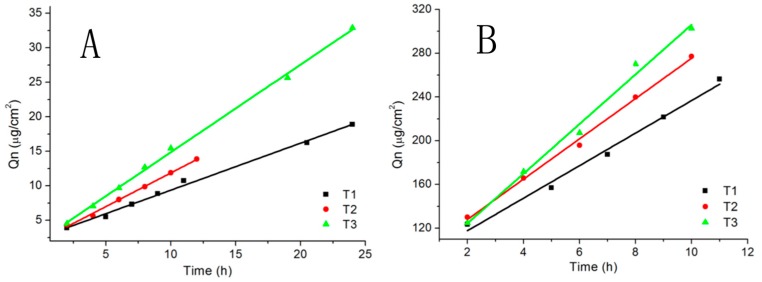
Cumulative amount of OST and BO at different temperatures: (**A**) OST; and (**B**) BO.

In the in vitro permeation studies, the concentrations of BO and OST were adjusted to 10.3743 and 0.7366 mg/mL (the concentration was set based on our previous experiment [22]). These studies were conducted at 298, 303 and 308 K. Figure 4 and Table 1 demonstrate the cumulative permeation amount of OST and BO, and *T_Lag_*, *J*_ss_, *K_pe_*, and *PR* of OST separately at different temperatures. Combined with the simulation results, with the increase of temperature, the lipid of the skin becomes looser, and fluidity increases so the permeability of the skin increases, which will help the drug through. In addition, the temperature increases, enhancing the molecular movement. There are more penetration enhancers (BO) penetrating into the skin more quickly, and the penetration ability is more significant, so the parameters *J*_ss_ and *Q_n_* also increase. The *T_Lag_* of OST at different temperatures can also prove that the higher the temperature, the faster the drug penetrates into the skin. In conclusion, the higher temperature can help BO enhance permeation of OST and help make OST cross more easily the bilayer raising the speed of permeation.

**Figure 5:**
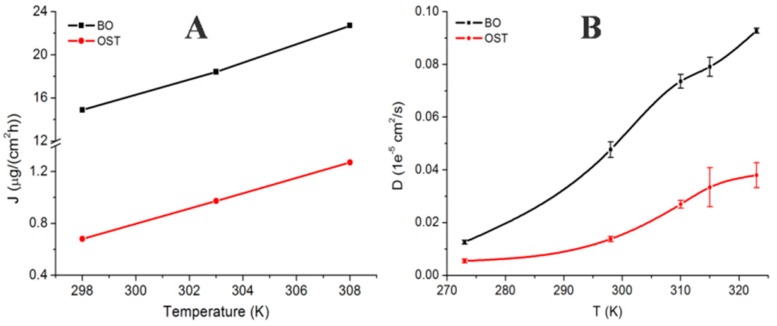
Diffusion rate of OST and BO at different temperatures: (**A**) steady-state transdermal flux (in vitro permeation studies); and (**B**) diffusion coefficient (in the coarse-grained molecular dynamic simulation).

To verify the consistency of the simulation, the diffusion parameter in the CG-MD simulation was compared with *J*_ss_ (shown in Figure 5); there was an obvious upward tendency in the two graphs, although they were not equal because of the deviation between the experiment and the simulation. In particular, at 298 and 310 K, the trends matched well, which certified the scientific basis of the CG-MD simulation.

#### 2.3.3. Transmission Electron Microscope Studies

**Figure 6:**
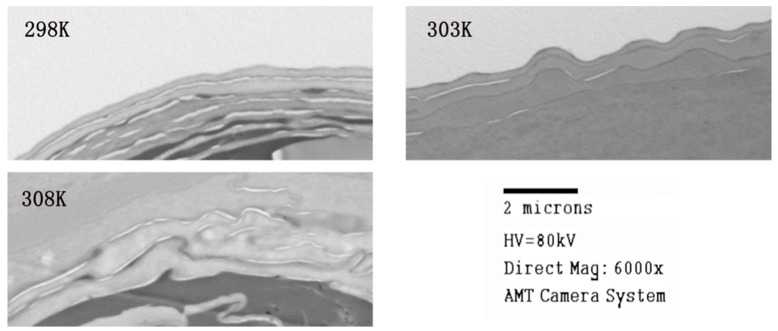
Transmission Electron Microscope (TEM) views of 24-h drug treated skin at different temperatures.

The skin samples were observed by TEM after the in vitro permeation studies. The skin samples were taken from in vitro permeation studies. Figure 6 shows the views of the skin at different temperatures; the bright stripe is the SC. At 298 K, the SC was orderly and smooth, but with the increasing temperature it was obvious that the SC became more disordered and rougher, and at 308 K, the SC were thoroughly destroyed. Hence, with the influence of BO as a permeation enhancer and appropriately increasing temperature, SC permeability became better. It is also worth noting that too much treatment destroys the bilayer structure as was the situation with 323 K and 5% BO.

## 3. Simulation Method

### 3.1. CG Models

**Figure 7:**
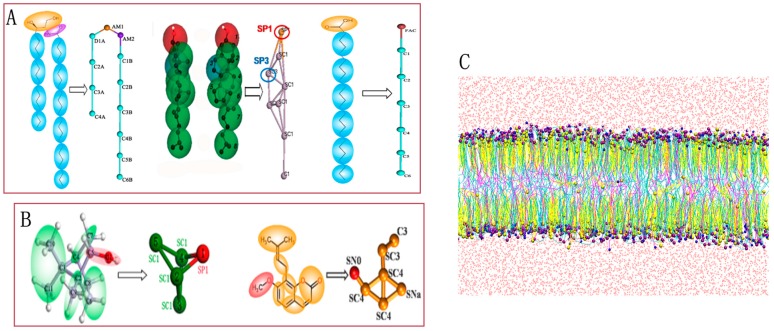
Chemical structure and coarse grained mapping for: (**A**) ceramides (CER), cholesterol (CHOL), and free fatty acids (FFA); (**B**) Borneol (BO), Osthole (OST); and (**C**) snapshot of blank membrane.

For the test case, we chose a compounded bilayer of ceramides (CER), cholesterol (CHOL), and free fatty acids (FFA) [41,42] in the ratio of 2:2:1. Our work mainly involved six molecules: CER, CHOL, FFA, BO, OST, and water. The lipid models (CER, CHOL, and FFA) and the solvent model W used in the simulations were downloaded from professional websites providing all of the resources about coarse-grain force fields for biomolecular simulations [43,44]. Figure 7A demonstrates the mapping method. Groups of four water molecules were replaced by a CG water bead using in-house scripts. Although the CG models of BO and OST were not provided by the website or other publications, they can be indirectly obtained as a standard script from the Marrink Group. This script is able to convert all-atom models to their corresponding MARTINI CG models. The bottom-up approach is a systematic parameterization strategy whereby data from AA simulation are used to determine the CG parameters, and the angle and bond parameters are set based on Boltzmann inversion. The mapping methods and parameters are shown as Figure 7B [45]. In the CG model of BO, a polar particle (SP1) was adopted to represent the hydroxyl group, while in the CG model of OST, the particle SN0 and SNa were selected to model the methoxy group and the ester group, respectively. The CG model had been verified in our previous study [22], the whole parameters and verification procress are shown in the Supplementary Materials (Figures S4–S7 and Tables S1 and S2).

The starting configuration for the mixed bilayer was constructed in a cubic simulation box with the dimension of 15 × 15 × 10 nm^3^. The 252 CER, 252 CHOL, and 126 FFA molecules in a molar ratio of 2:2:1 were placed in the bilayer model. A side view of a fragment of the blank bilayer is displayed in Figure 7C. The bilayer systems with different drugs in water were built by the Packmol software package (Institute of Chemistry and Institute of Mathematics, University of Campinas and Institute of Mathematics and Statistics University of São Paulo, Brazil) [46], and the figures depicting lipid molecules were produced by Visual Molecular Dynamics (VMD) [47].

### 3.2. Simulation Details

The simulation was conducted with the GROningen MAchine for Chemical Simulation (GROMACS, Ver. 4.6.3). Prior to simulation, the system was relaxed through energy minimization (EM) using the steepest descent algorithm through which the potential energy was descended to be negative on the order of 10^5^–10^6^ and the maximum force was adjusted to less than 80 kJ·mol^−1^. Standard simulation parameters associated with the MARTINI force field were used. The temperature was regularized constantly by using a Berendsen temperature coupling with a time constant of 1.0 ps, and the pressure was controlled by a Berendsen Barostat and semi-isotropic pressure coupling with a constant of 3.0 ps and compressibility of 4.5 × 10^−4^/bar. The neighbor searching algorithm was implemented and the cut-off distance was set as 1.4 nm. The method was shifted and the cut-off length was picked at 1.2 nm for both the Van der Waals and electrostatic potentials. A time step was preset as 20 fs, and finally, trajectory data with 400 ns in total was gained.

### 3.3. In Vitro Permeation Studies

#### 3.3.1. Materials and Reagents

Borneol and osthole (purity > 98%) were purchased from the National Institutes for Food and Drug Control (Beijing, China). Methanol and acetonitrile of HPLC grade were supplied by Thermo Fisher Scientific (Beijing, China). All other reagents of analytical grade were readily available from various commercial sources.

#### 3.3.2. Preparation of Osthole Solutions with Borneol

Fine osthole powder was carefully weighed and dissolved in 20/80 (*v*/*v*) water/propanediol. A piece of borneol weighing 26.10 mg was subsequently dissolved and mixed into the container and the solution was concentrated to 5.22 mg/mL borneol for preparation.

#### 3.3.3. Skin Preparation

The rats were anesthetized by excess ether inhalation, and the abdominal skin was excised after removing the hair with an animal hair clipper. After removing the fat and subcutaneous tissue, the skin was cleaned with ultrapure water and 0.9% sodium chloride. All animal experimental procedures were conducted in conformity with the institutional guidelines for the care and use of laboratory animals.

#### 3.3.4. Skin Permeation

Freshly-excised rat stratum corneum was immediately mounted over the modified Franz-type vertical diffusion chambers. Blank 80% propanediol (10 mL) was added to the endothelial side and maintained at 25, 30 and 35 °C under mixed conditions with 1 mL of donor solution of osthole and borneol with constant magnetic stirring, rotating at the speed of 300 rpm. The available corneal area for diffusion was 0.785 cm^2^. Samples of 2 mL were taken from the endothelial side and replaced with an equal volume of blank 80% propanediol at the time points: 2, 4, 6, 8, 10, 12 and 24 h. All of the solution samples were filtered through a 0.45-μm Millipore filter (Jin Teng Experimental Equipment Co., Ltd., Tianjin, China) and stored at 4 °C.

#### 3.3.5. Instrumentation and Chromatographic Conditions

A GC-FID system comprised of a HP-7890N gas chromatographic (GC) system and a flame ionization detector (FID) was applied in this study. Borneol was separated with a high-performance capillary column (30 m × 0.32 mm × 0.25 μm). Nitrogen, at a flow rate of 1 mL/min, was used as a carrier gas. The oven was programmed to rise from an initial temperature of 115 °C (maintained for 5 min) to 150 °C at the rate of 7 °C/min and held isothermally at 150 °C for 5 min, and then increased to 220 °C by post running for 10 min. The detector temperature was maintained at 300 °C and then 1 μL of samples were injected automatically [48].

The quantitative determination of osthole was performed with an HPLC system (Agilent 1100, Agilent, Inc., Santa Clara, CA, USA) using acetonitrile-water (65:35 *v*/*v*) in the mobile phase at a flow rate of 1.0 mL/min. The injection volume was 10 μL. A Waters Xbridge C18 column (250 mm × 4.6 mm, 5 μm, Waters, Inc., Hong Kong, China) was used. The UV detector (Waters, MA, USA) wavelength was set at 322 nm and the column temperature was maintained at 35 °C.

#### 3.3.6. Important Assessment Parameters

Skin permeation parameters, steady-state transdermal flux (*J*_ss_, μg/cm^2^·h), permeation coefficient (*K*_p_, cm/h), enhance ratio (*ER*), and permeation ratio (*PR*) were determined from the in vitro skin permeation data [49]. The cumulative amount (*Q_n_*, μg/cm^2^) of drugs permeated per unit area of skin is expressed by:

(1) $$Q_{n}=\frac{\left(C_{n}\times V_{r}+\sum_{i=1}^{n-1}C_{i}\times V_{i}\right)}{A},$$

where *C_n_* is the drug concentration of the receptor medium at each sampling time; *C_i_* represents the drug concentration at *i*th sampling point; *V_r_* and *V_i_* are the volume of receptor solution and sampling; and *A* means the effective diffusion area of skin. The relationship between *Q_n_* and *t* is plotted in the equation above. *J*_ss_ and lag time (*T_Lag_*) are extracted from the slope and X-intercept of the linear portion of the graph, respectively.

*K*_p_ was defined using Equation (2):

(2) $$K_{p}=J_{\mathrm{ss}}/C_{0}$$

*ER* and *PR* were calculated using Equations (3) and (4), with *V_d_* being the donor volume:

(3) $$ER=\frac{J_{\mathrm{ss}}\left(\mathrm{with PE}\right)}{J_{\mathrm{ss}}}$$

(4) $$PR=\frac{Q_{n}\times A}{C_{0}\times V_{d}}\times 100\%$$

#### 3.3.7. Transmission Electron Microscope (TEM) Studies

The skin samples were fixed instantaneously with 2.5% glutaraldehyde after permeation. Samples were then post-fixed in 1% OsO_4_ and dehydrated in a graded series of acetone. The samples were subsequently embedded in a low-viscosity epon-epoxy mixture and sectioned. Thin sections were double stained with uranyl acetate and lead citrate and then examined on a transmission electron microscope (JEOL JEM-1230, Tokyo, Japan) operated at 80 kV.

## 4. Conclusions

Temperature is a critical factor in transdermal penetration of drugs. Due to the influence of temperature on the transdermal penetration enhancing mechanism of borneol, we selected a multi-scale method, including CG-MD simulation and an in vitro permeation experiment.

In this project, the influence of temperature on the structure of the SC bilayer, the penetration of BO and OST, and the enhancing mechanism of BO to OST were gradually investigated. According to the structural parameters of the bilayer, it is obvious that rising temperatures could effectively enhance the permeability of SC. When the temperature increased, both BO and OST diffused into the bilayer quickly. BO is an effective penetration enhancer that promotes the permeation of OST by disturbing the ordered lipid to increase the bilayer permeability. With the effect of the increasing temperature and BO as a penetration enhancer, the bilayer’s morphological characteristics were changed drastically, which can effectively help strengthen the permeation enhancement of BO to OST. In addition, the in vitro permeation experiment demonstrated the influence of rising temperatures on the penetration enhancement ability of BO to OST by the permeation cumulative amount and *T_Lag_*, *J*_ss_, *K_pe_*, and *PR.* The TEM study provided a visual view of the SC changes. Furthermore, we also proved that the CG-MD simulation method can be a powerful tool for studying the permeation of SC or other bio-membrane systems.

## Figures and Tables

**Table 1:** Assessment parameters of Osthole calculated from *Q_n_*.

T (K)	*T_L__ag_* (h)	*J*_ss_ (μg/cm^2^·h)	*K_pe_* (cm/h)	*PR* (%)
298	3.7566	0.6811	0.0009	2.01
303	2.1817	0.9739	0.0013	2.22
308	1.6986	1.2704	0.0017	3.51

## Supplementary Materials

Supplementary materials can be found at www.mdpi.com/1422-0067/18/1/195/s1.
