# Supplementary material for: Influence of Temperature on Transdermal Penetration Enhancing Mechanism of Borneol: A Multi-Scale Study
Source: Int J Mol Sci. 2017 Jan 19;18(1):195. doi: 10.3390/ijms18010195 (PMC5297826; doi:10.3390/ijms18010195)
Supplement: Supplementary file 1 [file ijms-18-00195-s001.pdf]

Supplementary Materials: Influence of Temperature on Transdermal Penetration Enhancing Mechanism of Borneol: A Multi-Scale Study

Qianqian Yin, Ran Wang, Shufang Yang, Zhimin Wu, Shujuan Guo, Xingxing Dai, Yanjiang Qiao and Xinyuan Shi

1. Interaction of Different Concentrations of BO with SC Lipid Membrane

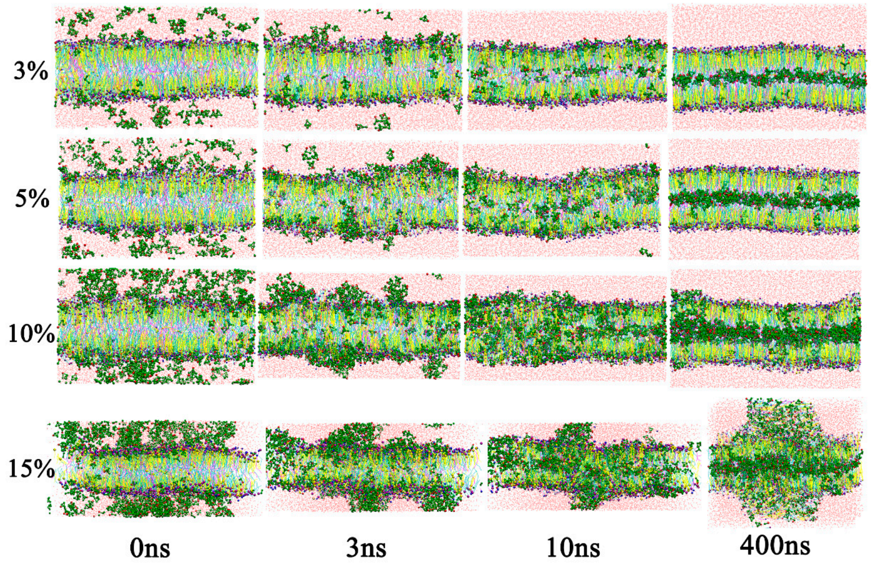

Figure S1. The morphology evolution of lipid bilayer systems with different concentrations of BO added during the simulation time.

2. Interaction of Different Concentrations of OST with SC Lipid Membrane

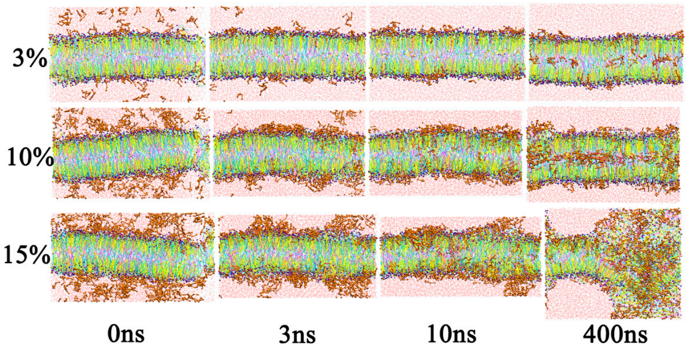

Figure S2. The morphology evolution of lipid bilayer systems with different concentrations of OST added during the simulation time.

3. Interaction of Different Concentrations of BO and 10%OST with SC Lipid Membrane

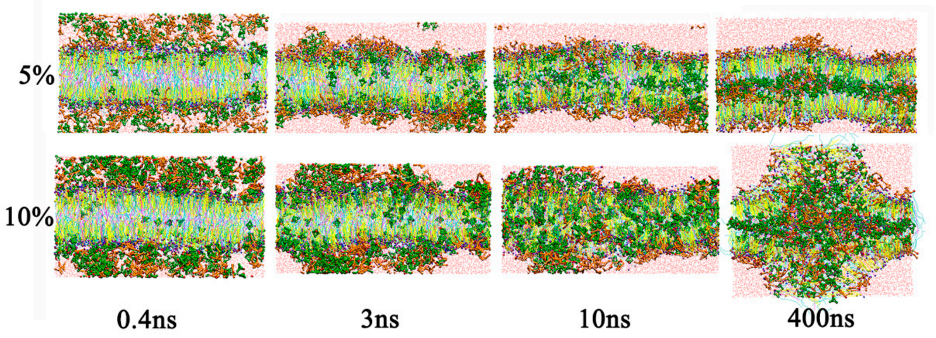

Figure S3. The morphology evolution of lipid bilayer systems with different concentrations of BO and 10% OST added during the simulation time.

4. Parameterization and Optimization of the CG Model

In this project, the main molecules involved in our simulation systems are ceramides NS 24:0 (CER NS), cholesterol (CHOL), free fatty acids 24:0 (FFA), borneol (BO) and osthole (OST). Among them, the Martini force field parameter for CER, CHOL and FFA were obtained from Marrink's study (see Figure S4 and Table S1).

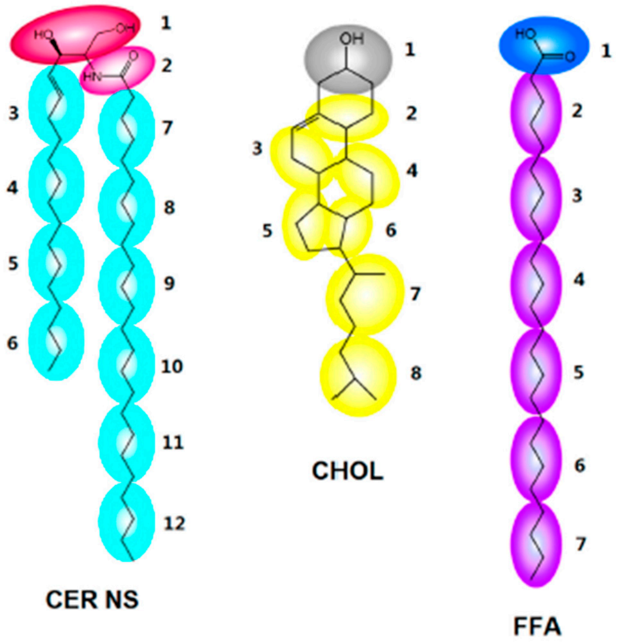

Figure S4. CG models of CER NS, CHOL, FFA.

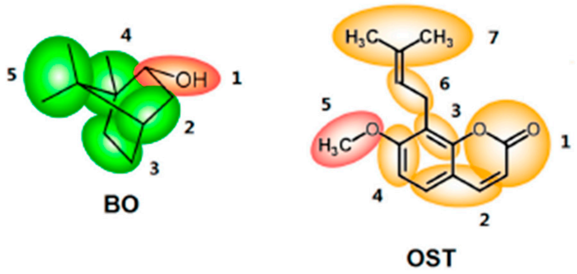

Figure S5. CG models of BO and OST.

Table S1. Force field parameters of CER NS, CHOL, FFA.

CER		
**Atoms** #	**Name**	**Type**
1	AM1	P4
2	AM2	P5
3	D1A	C3
4	C2A	C1
5	C3A	C1
6	C4A	C1
7	C1B	C1
8	C2B	C1
9	C3B	C1
10	C4B	C1
11	C5B	C1
12	C6B	C1
**Bonds** *i j*	**Length (nm)**	K_{bond} **(kJ·mol^{-1}·nm^{-2})**
1 2	0.27	20,000
1 3	0.29	20,000
3 4	0.47	1250
4 5	0.47	1250
5 6	0.47	1250
2 7	0.37	20,000
7 8	0.47	1250
8 9	0.47	1250
9 10	0.47	1250
10 11	0.47	1250
11 12	0.47	1250
**Angles** *i j k*	**Angle (deg)**	K_{angle} **(kJ·mol^{-1}·nm^{-2})**
7 2 1	129	200
1 3 4	180	25
3 4 5	180	25
4 5 6	180	25
2 7 8	180	25
7 8 9	180	25
8 9 10	180	25
9 10 11	180	25
10 11 12	180	25
**CHOL**		
**Atoms** #	**Name**	**Type**
1	ROH	SP1
2	R1	SC1
3	R2	SC3
4	R3	SC1
5	R4	SC1
6	R5	SC1
7	C1	SC1
8	C2	C1

Table S1. *Cont.*

CHOL		
**Bonds** *i j*	**Length (nm)**	**K_{bond} (kJ·mol^{-1}·nm^{-2})**
1 2	0.242	20,000
2 3	0.260	20,000
2 4	0.341	20,000
4 6	0.213	20,000
4 7	0.544	20,000
5 6	0.203	20,000
6 7	0.368	20,000
7 8	0.425	1250
1 3	0.493	constraint
1 4	0.604	constraint
3 4	0.272	constraint
3 5	0.346	constraint
4 5	0.294	constraint
5 7	0.406	constraint
**Angles** *i j k*	**Angle (deg)**	**K_{angle} (kJ·mol^{-1}·nm^{-2})**
4 7 8	180.0	25
**Impropers** *i j k l*	**Angle (deg)**	**$K_{impropers}$ (kJ·mol^{-1}·rad^{-2})**
1 3 5 4	0	100
1 3 5 7	0	100
1 4 5 3	0	100
3 5 7 4	0	100
4 7 5 3	0	100
2 1 3 4	−45	300
2 4 3 1	45	300
6 4 5 7	−45	300
6 7 5 4	45	300
**FFA**		
**Atoms** #	**Name**	**Type**
1	FAC	P3
2	C1	C1
3	C2	C1
4	C3	C1
5	C4	C1
6	C5	C1
7	C6	C1
**Bonds** *i j*	**Length (nm)**	**K_{bond} (kJ·mol^{-1}·nm^{-2})**
1 2	0.370	20,000
2 3	0.470	1250
3 4	0.470	1250
4 5	0.470	1250
5 6	0.470	1250
6 7	0.470	1250
**Angles** *i j k*	**Angle (deg)**	**K_{angle} (kJ·mol^{-1}·nm^{-2})**
1 2 3	180.0	25
2 3 4	180	25
3 4 5	180	25
4 5 6	180	25
5 6 7	180	25

Because no Martini force field parameter for BO and OST had been reported before, we parameterized it using method provided in Martini website. Their chemical structures and CG mappings are shown in Figure S5 and their force field parameters are shown in Table S2.

Table S2. Force field parameters of BO and OST.

BO		
**Atoms** #	**Name**	**Type**
1	BOH	SP1
2	BC1	SC1
3	BC2	SC1
4	BC3	SC1
5	BC4	SC1
**Bonds** *i j*	**Length (nm)**	K_{bond} **(kJ·mol^{-1}·nm^{-2})**
1 2	0.38	20,000
1 4	0.38	20,000
2 3	0.38	20,000
2 5	0.38	20,000
3 4	0.38	20,000
4 5	0.38	20,000
2 4	0.41	constraints
**Angles** *i j k*	**Angle (deg)**	K_{angle} **(kJ·mol^{-1}·nm^{-2})**
1 2 3	93.375	25
1 2 5	93.375	25
1 4 3	93.375	25
1 4 5	93.375	25
3 4 5	93.369	25
3 2 5	93.369	25
4 5 2	65.684	25
**Dihedrals** *i j k l*	**Angle (deg)**	$K_{dihedral}$ **(kJ·mol^{-1}·rad^{-2})**
5 2 4 1	120	100
5 4 2 3	120	300
3 4 2 1	120	100
**OST**		
**Atoms** #	**Name**	**Type**
1	OS1	SNa
2	OS2	SC4
3	OS3	SC4
4	OS4	SC4
5	OS5	SN0
6	OS6	SC3
7	OS7	C3

Table S2. *Cont.*

OST		
**Bonds** *i j*	**Length (nm)**	**K_{bond} (kJ·mol^{-1}·nm^{-2})**
4 5	0.43	1250
3 6	0.43	1250
6 7	0.43	5000
1 2	0.27	constraints
1 3	0.27	constraints
2 3	0.27	constraints
2 4	0.27	constraints
3 4	0.27	constraints
**Angles** *i j k*	**Angle (deg)**	**K_{angle} (kJ·mol^{-1})**
2 4 5	162.20	25.0
3 4 5	102.62	25.0
1 3 6	120.64	25.0
2 3 6	162.90	25.0
4 3 6	116.48	25.0
3 6 7	115.86	45.0
**Dihedrals** *i j k l*	**Angle (deg)**	**$K_{dihedral}$ (kJ·mol^{-1}·rad^{-2})**
1 2 4 3	0.000	50
5 2 3 4	0.553	50
6 2 4 3	9.113	50

Then, in order to validate the accuracy of our model, we calculated the bond distributions of molecules in all atoms (AA) and CG levels. The results shown in Figures S6 and S7 indicated that all the bond distributions of BO and OST in CG representations were similar to those in the AA representations, which demonstrated that our CG models are valid for further simulation studies.

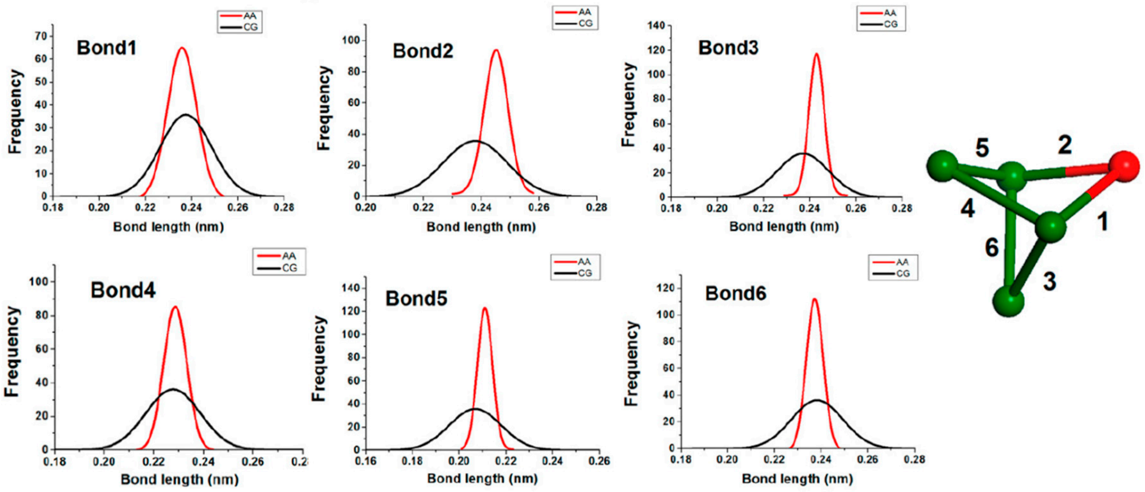

Figure S6. Bond distributions of BO molecules calculated from AA and CG simulations.

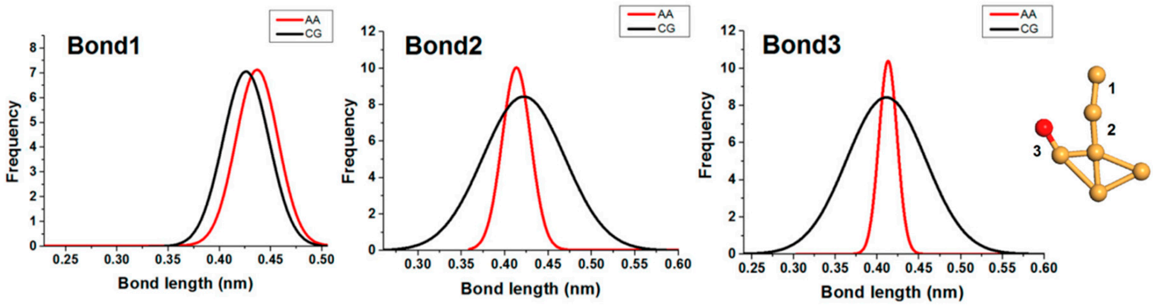

Figure S7. Bond distributions of OST molecules calculated from AA and CG simulations.
